# Predicting Kudzu (*Pueraria montana*) spread and its economic impacts in timber industry: A case study from Oklahoma

**DOI:** 10.1371/journal.pone.0229835

**Published:** 2020-03-16

**Authors:** Paulina Harron, Omkar Joshi, Christopher B. Edgar, Shishir Paudel, Arjun Adhikari

**Affiliations:** 1 Oklahoma State University, Stillwater, Oklahoma, United States of America; 2 University of Minnesota, St. Paul, Minnesota, United States of America; University of Molise, Isernia, ITALY

## Abstract

Quantifying the economic impacts of invasive species is an essential step in developing and prioritizing invasive species management. In particular, kudzu, *Pueraria montana* (Lour.) Merr. is an aggressive and non-native vine that not only causes ecological damage and reduces biodiversity, but can have multiple economic consequences such as loss of timber value and volume. Using current infestation locations in Oklahoma, southcentral USA, a Monte Carlo simulation was run to estimate the natural as well as anthropogenic spread rate of kudzu in the next five years. Simulations were supplemented with an economic impact analysis within the Impact Analysis for PLANing (IMPLAN) platform. To account for economic loss in the forest product industry, a replacement cost approach with a sensitivity analysis was conducted. Occurrence data collections revealed that current kudzu populations are already established in Oklahoma forests. The results demonstrate that by year five, total industry output could be reduced by $167.9 million, which will influence 780 jobs in the most extreme case scenario. The predicted economic loss due to kudzu expansion could act as an incentive for appropriate management practices and plans to be implemented.

## 1. Background

The rapid spread of non-native, invasive species is of growing concern in the United States (U.S.) and around the world. Invasive species have the potential to cause harm to the environment, the economy, and to human health [1]. In the U.S. alone, there have been a reported 50,000 invasive species that have been introduced, either accidentally or intentionally, in the past century [2]. Due to these infestations, annual economic costs have reached approximately $120 billion from damages and production losses [3]. Specifically, invasive weeds are accountable for an approximate $33 billion loss in crop production annually within the U.S., as well as contributing to an increase in herbicide control costs of about $4 billion [1]. Research has shown that the distribution of these species are on the rise as more introductions are occurring due to anthropogenic activities [4].

There have been efforts described in the literature highlighting the spread or immigration rate of certain invasive species. Research done by Williamson and Brown [5] focused on varying immigration models used to predict immigration rates of several invasive species in Britain, such as the muskrat (*Ondatra zibethicus*) and grey squirrel (*Sciurus carolinensis*). The authors found that many spread models are non-normal distributions with stochastic processes [5]. Lonsdale [6] focused on the rate of spread of *Mimosa pigra*, a woody weed, in Australian wetlands, in which the author demonstrated that rainfall heavily influenced seed dispersal and the population doubled in only 1.2 years within the river system. Although some research has envisioned spread and immigration rates of invasive species, economic analysis could further assess their broader impacts and management decisions.

Several studies have assessed the economic impacts of invasive plants using input-output analysis. For example, a study on yellow starthistle (*Centaurea solstitialis*) estimated an annual economic loss of $12.7 million (in 2005 dollars) in Idaho rangelands in association with this plant invasion [7]. In another study, Eiswerth et al. [8] estimated an annual loss of $5.9 million associated in management and damage costs due to the invasion of non-native grasses and weeds in Nevada. Liu and Piper [3] predicted the short-term spread of the invasive red streaked leafhopper (*Balclutha rubostriata*), which potentially could cause total economic costs of $5.4 to $71.6 million in Louisiana parishes.

One of the most threatening invasive species in the U.S. is *Pueraria montana*, also known as kudzu [9], which was introduced to the U.S. as a perennial vine during 1870s [10]. Since its introduction, kudzu has aggressively invaded over three million hectares across the U.S. and is estimated to spread at a rate of 50,000 hectares per year [11]. Kudzu’s aggressive characteristics result in a number of ecological impacts including shading out native species in forest understories [11], altering soil chemistry by fixing nitrogen in invaded soils [9], and decreasing native biodiversity [12]. In a recent study, Aurambout and Endress (2018) simulated kudzu spread utilizing its population dynamics and life history characteristics and reported that spread of kudzu due to seed dispersal could be six times higher than no seed dispersal and no early vegetative spread.

Invasive species such as kudzu not only poses ecological and environmental threats, but can adversely impact the economy as well as the aesthetic quality of natural resources [2, 11]. Research suggests that in the U.S. alone, the economic cost of kudzu totals as much as $100 million in damage annually due to lost productivity of the forestry industry, power and railroad companies, national and state parks, and agricultural land [9], as well as increased control and maintenance costs [13]. Forestry companies are paying approximately $500 per hectare per year for five years to control kudzu infestation [11, 14], while power companies are paying $1.5 million a year to manage kudzu and make up for power loss [11]. National and state parks are seeing a trend in reduced tourism due to declining aesthetic quality by widespread invasion of kudzu. [13].

An industry critical to the economy of Oklahoma is the forest products sector. In 2016, Oklahoma’s approximate 2.9 million hectares of timberland forests contributed $3.3 billion directly in terms of industry output in the state with total economic impacts that totaled up to $5.1 billion in production output, supported over 19,000 jobs and provided a payroll of $1.0 billion [15]. As an aggressive invasive species, kudzu has the ability to cause substantial losses to the forest products sector of the state. It can establish in both healthy and disturbed habitats with the ability to invade forest margins and create dense canopy mats on top of trees [9, 16]. The invasion of this species into forested areas in the past has resulted in forestry production losses of $100 to $500 million a year in the U.S. [11].

While spread rate and economic impact analysis have been done on a handful of invasive species, the economic effects that kudzu establishment and future expansion could have on timber markets, to the best of our knowledge, has yet to be investigated. Our research has a two-fold contribution to the existing knowledge. First, using a Monte Carlo simulation model, we have identified the vulnerable timberland regions, which are at risk of kudzu invasion across the State of Oklahoma. Second, we have quantified the potential economic impact of kudzu invasion on timber industries in Oklahoma, USA.

## 2. Methods

### 2.1 Spread rate model

Since the purpose of this IMPLAN analysis is to predict economic costs of kudzu based on its potential future expansion in Oklahoma, it is necessary to understand where kudzu could potentially spread based on current presence in the state. To this end, we identified 76 presence points in Oklahoma through the Oklahoma State University herbaria, Oklahoma Forestry Services records, Oklahoma State Vascular Plant Database, nursery and plant specialists, previous literature [16], databases such as EDDMapS 2018 and the Global Biodiversity Information Facility (GBIF), and citizen science such as iNaturalist. Kudzu observations were ground-truthed to ensure whether kudzu was present, controlled, or eradicated. Current occurrence data of Kadzu was subset for the area of Oklahoma with at least 25% timberland as defined by Forest Inventory and Analysis program.

Following Liu and Piper [3], we adopted the following model for spread analysis:

$$P(x)={exp}^{\left(A-Bx\right)}$$ Eq 1

In the equation above, *P(x)* represents the probability kudzu will spread from one location to the next in a five-year time frame, *x* is the distance between two locations measured in meters, and *A* and *B* are the parameters chosen to match the calculated probability of kudzu spreading a certain distance [3]. Concerning the spread of kudzu, communication with kudzu researchers and use of previous literature has resulted in the following values for spread probabilities [17, 18]. The probability that kudzu spreads 30 meters is 90%, while the probability of spreading 1,610 meters (1 mile) is 0.05%. Consistent with its life history characteristics, the 90% probability mimics a conservative scenario of vegetative spread through adult plants [19]. On the other hand, 0.05% probability captures an aggressive but rare situation of seed dispersal, which includes movement of seed as well as vegetative spread [19].

We used a Monte Carlo simulation in R environment to project potential occurrence probabilities. We divided the timberland region of Oklahoma into 0.5 km X 0.5 km gridded cells, which were classified as either presence (1) or absence (0) of kudzu based on current occurrence records. The simulation was run for 6,000 iterations to project probability of kudzu occurrence across study area during the next five years. We limited economic impact analysis for the next five years because input-output impact analysis, as a snapshot IMPLAN model, assumes constant returns to scale and cannot be used to capture economic losses in the long run [3].

### 2.2 Input-output analysis

Input-output analysis is used as a predictive mathematical representation of how a change, or shift, in the quality or quantity of one or more commodities in the economy can alter the output, employment, and income of industries within the region of interest [8, 20]. This analysis demonstrates the flow or linkage of products from an industry to other industries, government, consumers, and laborers [21]. Input-output analyses are static models, meaning they only look at the condition of the economy at one particular point in time, and thus are limited to a shorter time horizon [3, 22].

Specifically, the Impact Analysis for Planning [23] software utilizes direct effects from regional sectors to calculate indirect and induced effects for all other potentially connected sectors [24]. Specifically, IMPLAN runs input-output models to measure the fluctuation of sales through employment, labor income, and value added for specific sectors [23].

This is done using the following matrix equation:

$$X=(I-A{)}^{-1}*Y$$ Eq 2

where *X* is the total industry output, *Y* represents final demand, and *(I-A)*, known as the Leontief Inverse, is used as a multipliers matrix [21]. The matrix equation allows for exploring two multipliers; the first examines the relationship between direct and indirect effects, while the second, the social accounting matrix (SAM) multiplier, uses direct, indirect, and induced effects to understand how household spending and patterns of consumer demand can influence local economies [25].

When studying the total economic effects, it is important to assess the direct, indirect, and induced impacts. Direct effects are the immediate change in production of an economic activity due to a change in an activity [21]. For example, timber damage due to kudzu establishment and extension can result into lack of timber available for manufacturing. Indirect effects, which can be categorized as upstream or downstream, are the secondary industrial impacts that result from the use of goods or services provided by or provided to the industries that were directly affected [8, 21]. The indirect effect here would be that the lack of products available will result in companies experiencing reduced business and loss of jobs. Finally, induced effects are referred to as the “ripple” impacts that are a result of household spending patterns [7, 21, 26]. Induced effects include the decline in jobs and change in household income, as this will in turn alter the consumption behavior of employees and influence the flow of money back into local businesses, such as restaurants, grocery stores, insurance companies. Since previous research on invasive species impact is primarily relied on quantification of economic losses from directly affected industries [3], our efforts are likely to provide holistic picture by capturing impacts even with indirect and induced industries.

### 2.3 Kudzu invasion scenarios and sensitivity analysis

Since Monte Carlo simulations provided kudzu invasion within the probability distribution, we followed work done by Aurambout and Endress [19] to generate vegetative and seed dispersal scenarios. The first scenario (scenario A) involved the most aggressive invasion, which envisioned seed production by adults and seed dispersal as well as vegetative spread. The parcels with 1 to 25% probability of invasion were accounted for this scenario. The second scenario (scenario B) envisioned an aggressive vegetative spread via adults and saplings. Since vegetative spread by saplings is found to have a significant impact on kudzu’s capacity to invade [19], parcels with 25% to 100% probability of invasion were accounted for this scenario. Finally, we created a most conservative scenario (scenario C), which envisioned a vegetative spread via adult plants only. Since kudzu’s invasion capacity becomes minimal without seed dispersal [19], we assumed that this scenario could be mimicked by accounting for only 10 percent of the area under aggressive vegetative spread alone.

**Fig 1:**
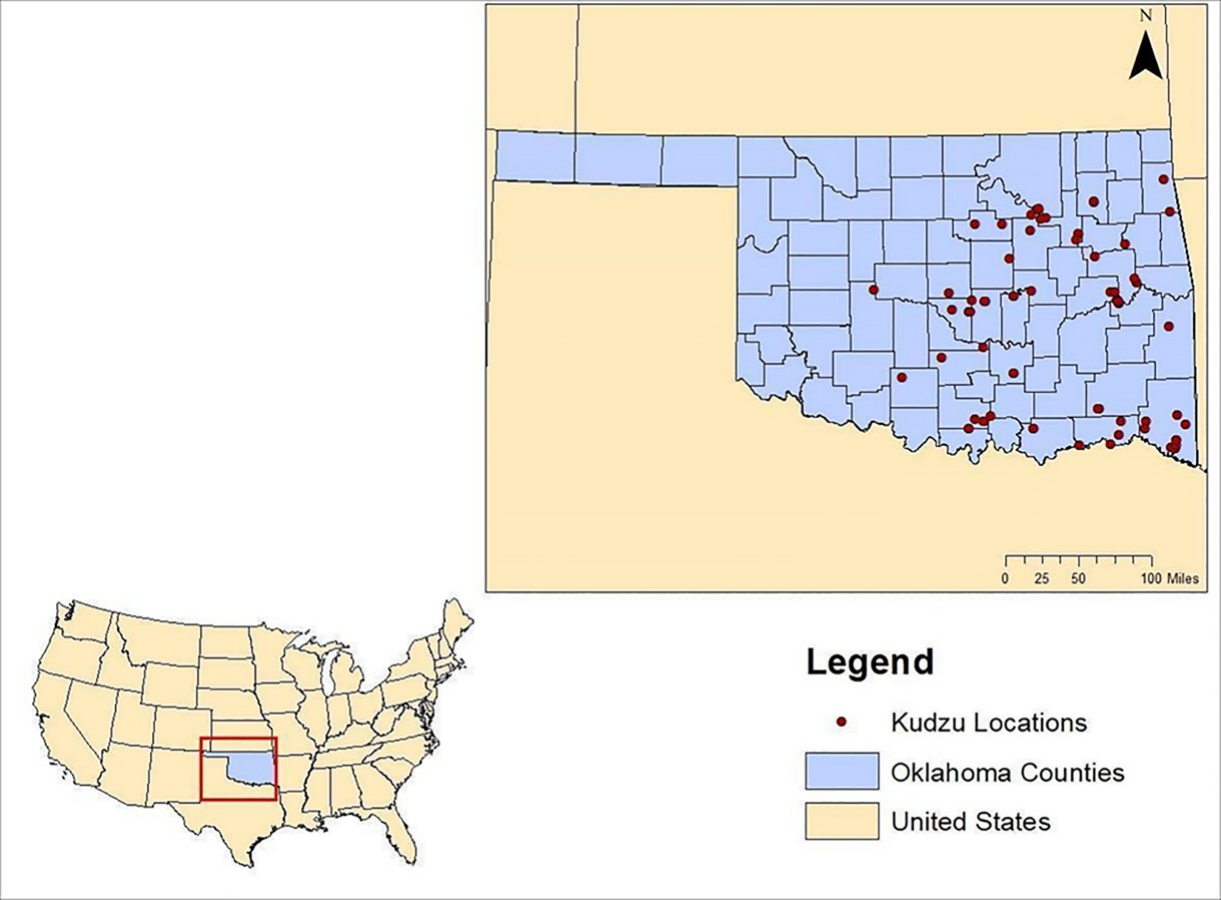
The 76 presence points of kudzu (*Pueraria montana*) collected in Oklahoma, USA, as of the Spring of 2018.

Existing literatures contradict on the reproductive behavior of kudzu, which influences its spread rate. For example, while some past studies have reported only thick roots of kudzu can survive colder weather conditions [27], study by McClain, Shimp [28] documented germination of kudzu seeds in Illinois. Although anecdotal, distribution of our known kudzu locations, as depicted in Fig 1, also suggests potential seed dispersal capacity of kudzu in our study region. Nonetheless, to account for inherent uncertainty with seed dispersal, we conducted a sensitivity analysis to capture how economic impacts may change, when kudzu dispersal becomes less aggressive than scenario A (i.e. 75% area of scenario A, 66% area of scenario A, and 50% area of scenario A).

**Analysis.** Using the results from the Monte Carlo simulations, we simulated the extent of kudzu spread in the next five years in timber producing regions of Oklahoma. Next, the number of infested cells that intersect timber regions were tallied up for each year of spread. Total economic impacts [15] of timberland area (~3 million hectares) were prorated for kudzu infested regions. Protocols for sector selection suggested by Joshi et al. [29] were used for economic contribution analysis. As documented in existing literature [e.g. 29, 30], loss in timber production impacts primary and secondary products, such as paper and paperboard products as well as solid wood products such as sawmills and pulp mills. The prorated impacts were then used as inputs for the IMPLAN model analysis. The protocol suggested in existing literature was followed [24, 29, 30]. For input-output analysis, the event years span from 2016 to 2020 as we used the latest 2016 IMPLAN data for the state of Oklahoma [23]. Additionally, all total economic results for both industries are reported in 2016 dollars. The predictions were made using 2016 Oklahoma IMPLAN data, which was the latest available until the initiation of this study. Therefore, five years period present the year 2016–2021, reported in 2019 US dollars.

## 3. Results

Monte Carlo simulation was based on the presence point identified in Oklahoma (Fig 1).

**Fig 2:**
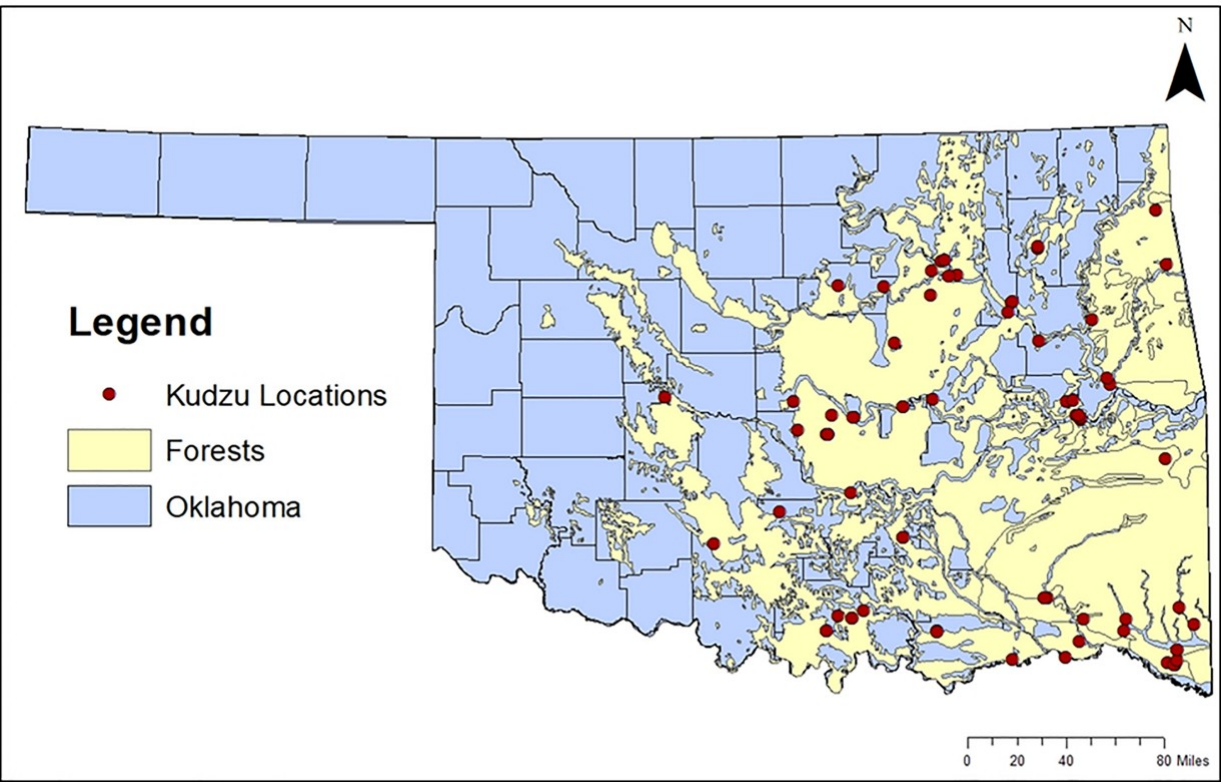
The confirmed 76 presence points of kudzu (*Pueraria montana*) as of Spring 2018 in relation to forest regions in Oklahoma, USA. The forest data layer was gained from the Game Types Map of Oklahoma digitized by Hoagland and Johnson [32] from the Oklahoma Biological Survey.

The Monte Carlo simulation showed that overall, kudzu can spread 104,464 acres in the first year and it was increased by ~185% occupying 297,144 acres across timberland of Oklahoma in the next five years. Results for all direct and total economic impacts, including employment and industry output, for all scenarios involving the timber industry are presented in Table 1. Currently in Oklahoma, there is about 2.7 million hectares of timberland [31]. From the most recent economic analysis, the forest sector contributed $3.3 billion directly to Oklahoma’s economy [15]. Fig 2 shows the extent of Oklahoma’s forests with the location of current kudzu presence.

**Table 1:** Cumulative total economic impacts for the timber industry under aggressive invasion of kudzu due to dispersal (scenario A). Results are reported in 2019 U.S. million dollars.

Impact Year	*Employment (No*. *of jobs)*	*Labor income (million $)*	*Value-added (million $)*	*Industry Output (million $)*
*Direct Impact*				
1	124	4.66	11.18	33.45
3	251	9.39	22.54	67.41
5	392	14.65	35.14	105.11
*Total Impact*				
1	248	10.79	21.56	53.42
3	500	21.76	43.45	107.66
5	780	33.95	67.76	167.89

The direct effects represent the direct value loss for the sum of all sector output values in each year of kudzu dispersal. Under scenario A, it is estimated that the timber industry experiences a loss of 124 jobs, $4.66 million in labor income, and $33.45 million in industry output directly in only the first year of spread (Table 1). After five years of spread, direct impacts reach $105.11 million in timber output loss, an effect on 392 jobs, and a $14.65 million reduction in labor income values. Moving to total economic impacts, from year one there are 248 jobs influenced, a $10.79 million change in labor income, and a devaluation of $53.42 million in the industry output. After five years of spread, and considering all impacts, we are seeing a cumulative change of $167.89 million in the value of this sector’s output, an effect on 780 jobs, and a $33.95 million reduction in labor income values (Table 1).

However, economic impacts under vegetative spread scenarios (scenario B and C), which reveal relatively lower spread rate of kudzu, are less severe (Table 2 and Table 3). For example, total economic impact due to invasion of kudzu due to vegetative spread by saplings (scenario B) will result into direct loss of 162 jobs and $43.46 million in economic output by year 5 (Table 2). Finally, economic impacts are minimal, if kudzu had a vegetative spread via adults’ plants only in scenario C (Table 3).

**Table 2:** Cumulative total economic impacts for the timber industry under invasion of kudzu due to vegetative spread by saplings (scenario B). Results are reported in 2019 U.S. million dollars.

Impact Year	*Employment (No*. *of jobs)*	*Labor income (million $)*	*Value-added (million $)*	*Industry Output (million $)*
*Direct Impact*				
1	76	2.83	6.79	20.32
3	157	5.87	14.08	42.12
5	162	6.06	14.53	43.46
*Total Impact*				
1	151	6.55	13.10	32.45
3	312	13.60	27.15	67.27
5	323	14.04	28.01	69.41

**Table 3:** Cumulative total economic impacts for the timber industry under slow invasion of kudzu due to vegetative spread by adults’ plants only (scenario C). Results are reported in 2019 U.S. million dollars.

Impact Year	*Employment (No*. *of jobs)*	*Labor income (million $)*	*Value-added (million $)*	*Industry Output (million $)*
*Direct Impact*				
1	8	0.28	0.68	2.03
3	16	0.59	1.41	4.21
5	16	0.61	1.45	4.35
*Total Impact*				
1	15	0.66	1.31	3.24
3	31	1.36	2.71	6.73
5	32	1.40	2.80	6.94

## 4. Discussion

In this study, we projected the aerial extent of kudzu spread across the timberland regions in the State of Oklahoma and its potential consequences in the economy over the next five years. Our simulation results, in an event of aggressive seed dispersal, revealed the pervasive infestation of kudzu across timber regions of SE Oklahoma. Multiple biotic (e.g. vegetative growth, seed dispersal) and abiotic factors (changing climate, disturbance regime) [33] could be responsible for the dramatic increase of kudzu infestation. Taking into account all of the plausible factors, kudzu infestation increased by ~185% in the next five years, which could have serious impacts on environmental and economic services of Oklahoma timberland.

Results reflected in the timber industry show that the current presence of kudzu and its potential expansion in Oklahoma’s forest can have substantial detrimental economic costs to the value of timberland and subsequently, to industries, employees, and consumers that rely on this industry. Over a short-time period, the future value of timber could decrease if management efforts are not taken and this can have significant implications on the $5.1 billion worth timber industry of Oklahoma [15]. Not to mention, some of these impacts could be seen in the decrease of timber products, a decrease in output by primary wood product producers and other manufacturing companies, and lack of marketable timber and income for private landowners.

These results could have several management and policy implications. First, as is evident from our analysis, the potential impacts of kudzu could be highly detrimental to timber companies, thus a few best management practices (BMP) can help control current populations. While one-size-fits-all formulas may not work in BMP implementation, past research encourages mechanical or herbicidal spraying based on the patch size and age, as well as the habitat being infested [34, 35]. Second, while reactive practices such as mechanical removal or herbicidal spray can eradicate kudzu invasion, any form of active land management practices come at a cost to the landowners [36]. Therefore, emphasis on BMPs that prevent the introduction and promote early detection of invasive species are the best cost-effective approaches for the sustainable management of non-natives [37]. In addition to information on preventive and reactive BMPs, information of potential future costs can act as an incentive to bring awareness to kudzu and limit its expansion. The appropriate outreach response from federal and state agencies, as well as university Extension programs, are recommended. Third, if future research is conducted on stakeholder perceptions of kudzu and willingness to pay (WTP) for BMPs, management plans can be more targeted and may benefit all involved parties. Results from this research can inform stakeholders of potential economic impacts and encourage them to seek out different BMPs, while future stakeholder analysis studies can provide a clear understanding of where management plans should begin for each stakeholder group based on WTP.

Fourth, while results suggest that kudzu does not seem to reach the northern areas of Oklahoma, we cannot rule out such possibility in the future. It is worth noting that kudzu is a carrier of soybean rust *(Phakopsora pachyrhizi* or *P*. *meibomiae)*—a disease introduced to the U.S. in 2004 [38]. Soybean rust is a fungus that attacks legume foliage leading to leaf lesions, early defoliation, and reduced pod production [39]. Kudzu leaves act as vectors for soybean rust, allowing it to survive winters and infect soybean crops in the spring [39]. Researchers have reported that soybean rust has resulted in soybean yield reductions of 30 to 80%, which corresponds to production losses of $640 million to $1.3 billion annually in the U.S. [40]. Looking specifically in the state of Oklahoma, soybean farms are an important component of the state’s economy. Although the majority of soybean farms are found in the northern part of the state, there is a potential for future expansion of kudzu into these regions and can put the soybean industry at severe risk of production loss. While our analysis did not take this into account, public awareness on potential economic impact of Kudzu on soybean industry is needed.

Fifth, this research can act as an incentive to begin a discussion about not only Oklahoma’s noxious weed list, but all states. Currently, kudzu is only listed on a handful of state noxious weed lists [41]. Without listing kudzu as a noxious weed, the transportation and growth of kudzu is not regulated, as well as there being no liability for controlling kudzu on personal property. Without this policy framework for most states, not much is being done to slow the movement of kudzu. Additionally, the establishment of kudzu in more northern areas, beyond its current distribution range, is very likely [33, 42]. Kudzu has demonstrated a strong tolerance for cooler climates [9, 43, 44]; its ability to establish in areas with larger soybean or timber industries can have both greater economic and ecological impacts than were estimated in this research. Finally, the study area represents a unique gradient of forest and open rangeland that allows the economic framework of this research to be mimicked for other forested or rangeland regions.

Finally, our study results made two methodological contributions to existing literature. First, similar to Kudzu, invasion from other invasive species (e.g.: Tallow tree, Tree-of-Heaven, Japanese Honeysuckle, Chinaberry tree, etc.) has resulted in significant timber value loss in the southern United States [45]. Therefore, the economic impact analysis of timber loss—the method used in our research—is readily applicable to other invasive species of interest. Second, the timber industry in Oklahoma is dominated by the pine forests [46], which are the most economically important forest types in the southern United States [47]. As such, social accounting multipliers capturing the relationship between total and direct economic losses (jobs, value-added, output, labor income) from this study are applicable to other kudzu-impacted pine forests having similar economic realities. To this end, landowners, land managers, and government agency professionals from other southern states can utilize our findings for potential economic impact analysis.

A few caveats of this study are worth noting. First, although there is documentation on kudzu invasion in SE Oklahoma, we could not find real data that could be used to gauge its potential economic impacts in the region. Also, as revealed from our analysis (Table 4 and Table 5), the direct and total economic losses in terms of outputs, valued-added, and the jobs substantially change depending upon assumed land area under kudzu invasion. Since the reproductive as well as seed dispersal behavior of kudzu is still not clear [19] and our simulation is based on several assumptions, we have provided possible aggressive and passive scenarios of kudzu invasion. Finally, while economic projections from input-output analysis provide broader societal impacts beyond a directly affected sector, they are limited for five years due to uncertainty of structural changes in economy [48]. Therefore, readerships are advised to make a cautious interpretation of our study results.

**Table 4:** A sensitivity analysis showing effect of uncertainty in kudzu dispersal rate on overall economic impacts for year 1.

Assumed area	*Employment (No*. *of jobs)*	*Labor income (million $)*	*Value-added (million $)*	*Industry Output (million $)*
*Direct Impact*				
50% area of scenario A	62	2.33	5.59	16.73
66% area of scenario A	83	3.10	7.46	22.30
75% area of scenario A	93	3.49	8.39	25.09
*Total Impact*				
50% area of scenario A	124	5.40	10.78	26.71
66% area of scenario A	165	7.19	14.37	35.61
75% area of scenario A	186	8.09	16.17	40.06

**Table 5:** A sensitivity analysis showing effect of uncertainty in kudzu dispersal rate on overall economic impacts for year 5.

Assumed area	*Employment (No*. *of jobs)*	*Labor income (million $)*	*Value-added (million $)*	*Industry Output (million $)*
*Direct Impact*				
50% area of scenario A	196	7.33	17.57	52.56
66% area of scenario A	261	9.77	23.42	70.07
75%area of scenario A	294	10.99	26.35	78.83
*Total Impacts*				
50% area of scenario A	390	16.97	33.88	83.94
66% area of scenario A	520	22.63	45.17	111.93
75%area of scenario A	585	25.46	50.82	125.92

## 5. Conclusion

The results from this study suggest that kudzu has invaded the south-eastern region of Oklahoma. Although kudzu has not reached the northern region of the state, it may invade northern soybean farms due to seed dispersal in the future. In timberland regions, the reduced timber availability, which serves as input for several timber products industries, may impact their supply chain dynamics. Additionally, these economic effects will have broader societal impacts and they affect employees who will have to suffer layoffs or alter their spending behaviors. Finally, while the economic impacts estimated in this study are for the five year period, they are expected to prompt discussion of management actions that can help control further spread of invasive species, including kudzu.

## Supporting information

S1 Data (XLSX)

S2 Data (TXT)
